# Risk of Dehydration Due to Sweating While Wearing Personal 2 Protective Equipment in COVID-19 Clinical Care: A Pilot Study

**DOI:** 10.3390/healthcare10020267

**Published:** 2022-01-29

**Authors:** Andrés Rojo-Rojo, Maria José Pujalte-Jesús, Encarna Hernández-Sánchez, Rafael Melendreras-Ruiz, Juan Antonio García-Méndez, Gloria María Muñoz-Rubio, César Leal-Costa, José Luis Díaz-Agea

**Affiliations:** 1 Intensive Care Unit, Hospital Virgen de la Arrixaca, Murcian Health Service, 30107 Murcia, Spain; arojo@ucam.edu (A.R.-R.); Gmmr1980@hotmail.com (G.M.M.-R.); 2 Faculty of Nursing, Universidad Católica de Murcia (UCAM), 30107 Murcia, Spain; ehsanchez@ucam.edu (E.H.-S.); cleal@um.es (C.L.-C.); 3 Maternity Ward, Hospital de Torrevieja, 03186 Alicante, Spain; 4 Faculty of Telecommunications Engineering, Universidad Católica de Murcia (UCAM), 30107 Murcia, Spain; rmelendreras@ucam.edu; 5 Emergency Management of the Region of Murcia, Murcian Health Service, 30005 Murcia, Spain; jagmendez@ucam.edu

**Keywords:** personal protection equipment, COVID-19, simulation, sweating, dehydration

## Abstract

Objective: The objectives of this study were (a) to determine the physical impact of the personal protective equipment (PPE) used in COVID-19 care, specifically the impact on the hydration state of the temperature and the comfort of the healthcare workers who use it, and (b) to show the high-fidelity simulated environment as an appropriate place to test the experimental designs to be developed in real environments for COVID-19. Background: All healthcare staff use full PPE in the care of COVID-19 patients. There are problems, such as excessive sweating, which have not been quantified thus far. Methods: A descriptive pilot design was used in a simulated high-fidelity setting. There was paired activity, with mild–moderate physical activity, between 45 and 60 min continuously, with the COVID-19 PPE. Sixteen intensive care nurses were selected. The before–after differential of weight, thirst, weight use of the PPE, body temperature, thermal body image, general and facial warmth sensation, and perspiration sensation were measured. Results: All subjects lost weight in the form of sweat with both PPEs during the simulation scenario, with a mean of 200 g (0.28% of initial weight), and increased thirst sensation. Body thermal image increased by 0.54 °C in people using the full COVID-19 PPE. Conclusions: The use of PPE in the management of critically ill COVID-19 patients generates weight loss related to excessive sweating. The weight loss shown in this pilot test is far from the clinical limits of dehydration. The use of ventilated PPE, such as PAPR, reduce the body temperature and heat sensation experienced by the users of it; at the same time, it improves the comfort of those who wear it. The simulated environment is a suitable place to develop the piloting of applicable research methodologies in future studies in a real environment.

## 1. Introduction

The COVID-19 pandemic we have been suffering since the beginning of January 2020 has profoundly changed how society, in general, and health professionals, in particular, provide care to hospitalized patients.

Despite the negative manifestations derived from the impact of COVID-19 on health systems, facing this pandemic has taught us a series of lessons for this and other pandemics to come [1]. Among these lessons, the use of personal protective equipment (PPE) by all professionals involved stands out, especially in critical hospital environments (intensive care units (ICUs), resuscitation units, emergency departments (EDs), etc.).

All health workers use PPE to protect themselves against physical contact, respiratory droplets (aerosols), and airborne transmission [2,3]. This implies the use of respiratory protection devices, such as filtering half-masks for protection against particles, with a filtering efficiency (according to standard UNE-EN 149: 2001 + A1:2010) of 92% (FFP2) for processes with a low risk of aerosolization or with a filtering efficiency of higher than 95% for processes with a high risk of aerosolization (FFP3 or N95), eye protection against aerosols and splashes (goggles and/or face shields) [4] and protective clothing. This clothing consists of a long-sleeved gown with adjustable cuffs that is waterproof, for the general care of confirmed COVID-19 patients, and increasing this protection with a hood and/or boot covers, or a full bodysuit in the case of a positive COVID-19 patient with “unpredictable” behavior, where activities are carried out under potential aerosolization [4,5].

Different studies [6,7,8] have confirmed that health workers in critical environments (especially ICUs) spend long periods of time with this equipment, lasting around eight hours on average, distributed in different periods throughout 12 h days. 

While wearing this equipment, different physical side effects have been described, such as headaches, discomfort, and heat stress, including the presence of wounds on the face (especially the cheeks and the bridge of the nose) [7,8,9]. Seventy percent (70%) of the subjects studied in previous research indicated the presence of excessive sweating after activity with PPE in a COVID-19 environment [10].

Experimental studies have assessed the impact of the use of PPE on professionals but in settings that are far different from the clinical environment, using very low-fidelity activities, without the main objective of measuring the impact of one’s own use of PPE [11,12].

The impact of physical activity on the state of dehydration–hypohydration has been widely studied and evidenced in the field of sports science and medicine. This indicates that the simplest method to evaluate the acute change in individuals is to compare their weight and/or body mass index (BMI) after using PPE with the initial values [10,13].

Clinical dehydration is considered to occur when losses greater than 1% of initial body weight are observed. The sensation of thirst is produced by an increase in plasma osmolarity, among other factors, which is indicative of dehydration. Various studies [14,15] have associated dehydration with losses greater than 1% with a loss of cognitive and/or motor capacity.

The aims of this study were (a) to determine the physical impact of the protective equipment used in COVID-19 settings, specifically the impact on the hydration state from temperature and the comfort of the healthcare workers who use it, and (b) to show that the high-fidelity simulated environment is an appropriate place to test the experimental designs to be developed for real environments for COVID-19. 

The impact was measured after wearing the two most-commonly used COVID-19 PPEs: Conventional PPE (PPE-Conv) and powered air-purifying respirator PPE (PPE-PAPR). Both types of PPE are described in the next section. Therefore, we measured the hydration status through weight loss before and after the simulated scenario.

## 2. Materials and Methods

### 2.1. Study Design 

The study was conducted using a descriptive pilot study design in a Zone 3 [16] simulated scenario. Simulation in “Zone 3” is a concept used to describe the performance of a simulated practice by health professionals in a nonclinical environment comparable to a clinical one. Roussin and Weinstock [16] used this nomenclature at the organizational level for the different types of simulations with which health professionals are trained and the different resources and methodologies to be used. They staged the simulation in five large areas (0–4), depending mainly on the environment and the students who perform the simulated scenes.

### 2.2. Setting and Sample

The sample study consisted of nursing professionals (registered nurses (RNs)) from critical care units who performed their professional activity in the ICU caring for critically ill COVID-19 patients in third-level Spanish hospitals of the Public Health Service, from March 2020 to the present, and had worn PPE when performing their care activities. 

The participants were selected by a convenient sampling strategy in the two hospitals, which were of the third level in the region, with 100 and 60 nursing professionals associated with them, respectively, using “key informant” in the units of origin of the nurses. 

All of the people selected were volunteers who, in their free time, went to the UCAM facilities to carry out the simulation scenarios. They spent approximately three hours in this activity. No reward was provided. 

As inclusion criteria, nursing professionals who formed a work team in their work unit were selected. This way, thirty (30) nurses were initially selected. Some of them did not attend the experiments for personal reasons; thus, the number of samples was lower than initially expected. The exclusion criteria were those situations that contraindicated activities in a critical COVID-19 environment. 

The final sample consisted of sixteen (16) RNs. Previous studies [11,12,17,18] have also included a similar number as the study sample. The RNs performed the scenario two times—once with PPE-Conv and another time with PPE-PAPR. Healthcare assistants (HCAs), who usually worked alongside the participating RN teams, participated as companions. 

The study took place between June and July 2021 in the Catholic University of Murcia’s (UCAM) simulation rooms.

### 2.3. Ethical Considerations

The Ethical Committee of the Catholic University of Murcia (report CE06211) approved this study. The study did not require a review by a clinical research ethics committee, as the research only involved nursing staff as participants. Verbal and written informed consent were obtained from the individuals who participated in the study. All of the participants were informed about the risks to their health derived from the experimental design, and all provided explicit consent for their participation. The expression “REAL END” was devised as an emergency key to suspend the simulation by the participants for any reason.

### 2.4. Simulation Room Setting

**Figure 1:**
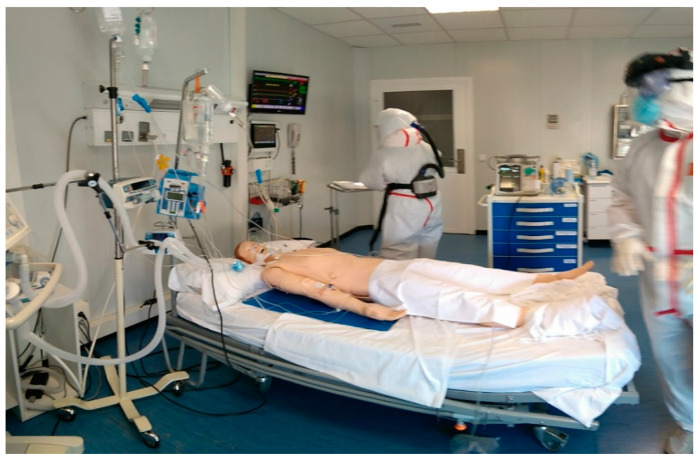
Simulation setting.

A simulated critical care unit environment was designed, which provided a high-fidelity setting. The room was equipped with medical equipment, consumable material and an infrastructure similar to that used in a real context (Figure 1). The environmental conditions of temperature (25 °C), humidity (60%), and noise (60 dB) were also replicated.

The simulator used was the Laerdal SimMan 3G^®®^ (Laerdal Medical, Stavanger, Norway) in which peripheral and central venous catheters, an arterial catheter, an orotracheal tube, bladder and nasogastric tubes and pressure ulcers in the sacral zone were installed.

### 2.5. Simulated Scenario Design

A clinical scenario was designed where basic care had to be performed on the simulated critical patient, for at least 30 min, after which a situation of clinical deterioration with cardiorespiratory arrest had to be tended to for no less than 15 min, which implied a minimum of 45 min of activity, and a maximum of 60 uninterrupted minutes. The activities are listed in Table 1. The scenario was designed by an expert instructor in clinical simulation and critical care. 

Each professional (RN and HCA) played a role according to the functions and competence assumed in their daily practice.

### 2.6. PPE Used

The different types of PPE used in the study are described in Table 2. All of the participants wore joint protective equipment (JPE): disposable soft surgical medical uniform; full-body suit protection, and hand (gloves) and foot protection. Table 2 includes an image of a subject with joint equipment before the experiment. The PPE-PAPR equipment combines respiratory protection and facial protection. 

### 2.7. Experimental Procedure

The scenario was carried out twice, separated by a minimum of 48 h. Each experiment reproduced the same conditions and the simulated scenario. The assignment of PPE for the RN in the first scenario was carried out randomly; the PPE used in the second scenario was the one not used in the first scenario. The HCA always wore the same protective clothing and did not take part in the measurement. The experimental protocol is detailed in Table 3. 

### 2.8. Data Collection Instruments

An ad hoc instrument was used, composed of three differentiated parts: (A) sociodemographic form; (B) observational form/anthropometric values pre/post and weight and PPE used pre/post; (C) Schumacher Comfort Scale, Borg Perceived Effort Scale and Fanger General Heat Scale.

The following variables were recorded with the different scales for each subject and each PPE: BMI; differential personal weight (DPW) after–before; percentage of weight lost (DPW × 100/PW after); differential weight joint protective equipment (DJPEW) after–before; differential thirst (DT) after–before; general heat sensation; sensation of facial heat; sensation of perspiration; personal body temperature (BT); and facial/body thermal image (FTI/ BTI). 

**Figure 4:**
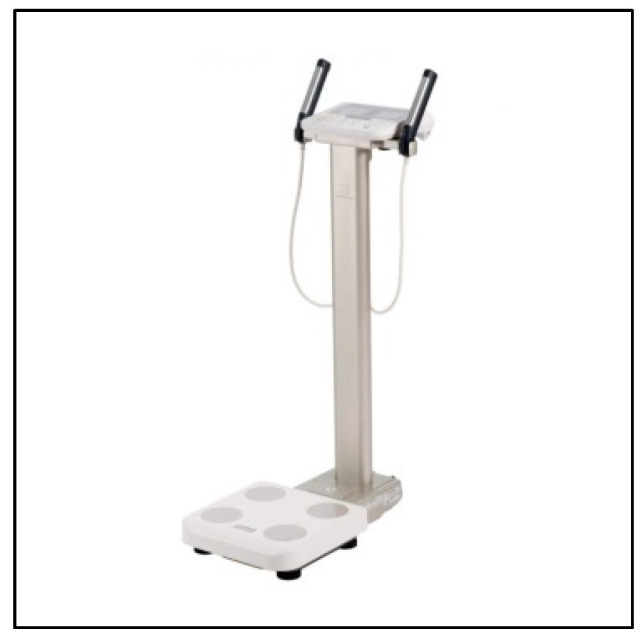
Tanita MC-780 P MA^®®^.

The weight of the participants was determined with the Tanita MC-780 P MA^®®^ scale (equipment accredited as Accredited MDD Class III and NAWI IIa), with precise intervals of 100 g (Figure 4).

**Figure 5:**
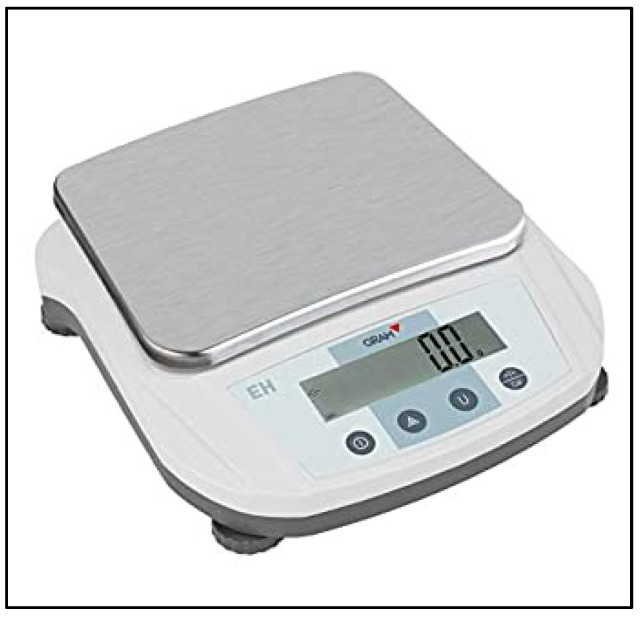
Gram EH6000^®®^.

The weight of the joint equipment was measured with the Gram EH6000^®®^ precision scale, with precise measurement intervals of 0.1 g (Figure 5).

The sensations of thirst, general heat, facial heat, and perspiration were determined with Likert-type scales, graded from less to greater intensity with values ranging from 0 to 5.

**Figure 6:**
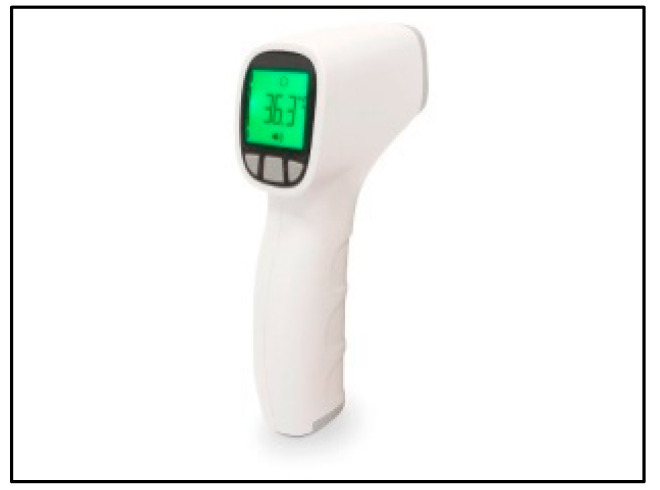
Mobiclinic-TO-01^®®^.

**Figure 7:**
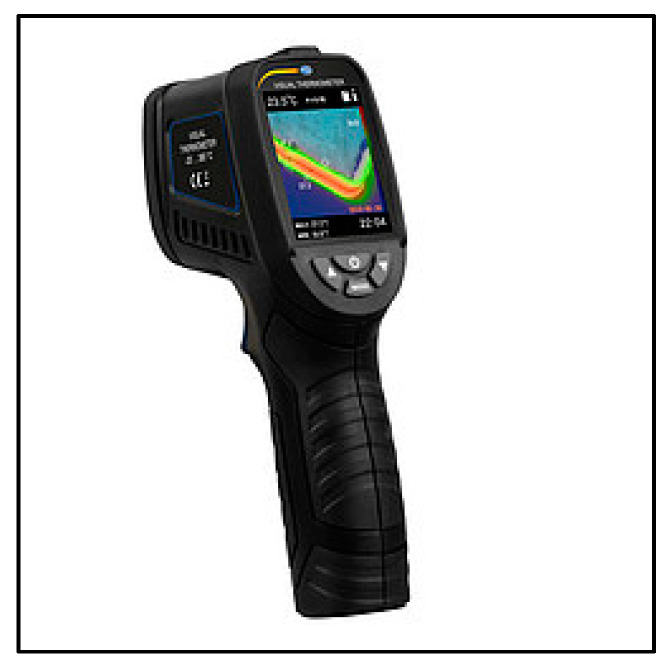
PCE-TC 24^®®^.

BT was measured before starting and after finishing the scenario. It was measured in the temple area with a contactless infrared thermometer (Moviclinic-TO-01^®®^, Mobiclinic Technology Co, Sevilla. Spain) (Figure 6). The face/body frontal thermal image was measured after the simulated scenario began (before 15 min acclimation) and just before the simulation scene, at 3 m (9 feet), using an infrared thermometer-camera PCE-TC 24^®®^, (PCE Holding GmbH-PCE Iberica LTD, Albacete. Spain) (Figure 7).

### 2.9. Data Analysis

The analyses were performed using MS Office Excel 2016^®®^ and IBM SPSS^®®^ version 21.0 software for Windows. A descriptive analysis of the data was carried out, where continuous variables were expressed as the mean and standard deviation (SD). Moreover, the asymmetry (skewness), kurtosis, and Shapiro–Wilk test were used to ensure the statistically normal distribution of the data. The differences between the variables were analyzed with Student’s test. The data were considered statistically significant at *p* < 0.05. Furthermore, the Pearson’s correlation coefficient (*r*) or Spearman’s rank correlation coefficient (*r*_s_) was used to compare the relationships between the different quantitative variables.

## 3. Results

Sixteen experimental subjects were included in this study. The sociodemographic and anthropometric values (age, gender, experience, hospital of origin, type of PPE used in the ICU, maximum length of time of continuous PPE wear, and BMI) are shown in Table 4.

### 3.1. Change in the Weight and Comfort Parameters

The personal weight (PW) and joint PPE weight for the groups (PPE-Conv and PPR-PAPR) were measured before and after the scenario. The thirst sensation was measured before/after. The height was measured before the beginning of the first scenario. The BMI before/after was calculated using the formula weight (kg)/height (m)^2^. 

Other comfort parameters were measured before scenario. The change in the weight and comfort parameters are shown in Table 5. 

The measured DPW (PW after–PW before) was around 200 g in the subjects that wore the PPE in both groups; it supposed a mean loss of 0.28% of the PW measured before the simulated scenario in both groups.

The DJPEW (JPEW after–JPEW before), increased in both groups: a mean of 75.75 g (SD = 57.62) for PPE-CONV and 65.06 g (SD = 45.96) for PPE-PAPR. If we disaggregate the PPE-W data taken with the PAPR subjects who wore it inside (PPE-PAPR-i) of the suit, or those who wore it outside (PPE-PAPR-o), the PPE-PAPR-i group had the lowest measurement: PPE-PAPR-i weighed 36.07 g less than PPE-PAPR-o.

To determine whether there was a significant difference between the PW of the participants recorded before and after the scenario and the DJPEW that the participants wore during the scene, we considered performing a paired *t*-test. They were measured in both PPE groups.

To do this, we asked ourselves the following question: “Is there a significant difference between the weight recorded before the simulated scenario and after it?” and determined the following hypotheses:

**Hypotheses** **0** **(H0).** *The average weight (PW or JPEW) level before the scenario is equal to the weight after it.*

**Hypotheses** **1** **(H1).** *The average weight (PW or JPEW) level before the scenario is different to the weight after it.*

We would accept H0 if the *p*-value was higher than 0.05 points; we would accept H1 if the *p*-value was lower than 0.05 points. 

The paired *t*-test data are shown in Table 6.

The significance or *p*-value (sig.-bilateral) was 0.000 < 0.05; hence, we can conclude that there was a significant difference between the weight before and after the scenario; thus, we can infer that wearing PPE in any of its types reduces the weight of those who wear it. 

The thirst sensation increased in all participants. They increased their differential of thirst by a mean of 2.5 points (this measure being a little higher in PPE-Conv than in PPE-PAPR).

The other comfort sensations (facial heat, general heat, and perspiration) were better in the PPE-PAPR than the PPE-Conv group. 

### 3.2. Change in the Temperature Parameters

The change in the temperature of the participants is shown in Table 7. 

The BT recorded at the temple zone of the participants underwent slight changes according to the PPE used, without assuming dangerous limits for health. Thus, those who used PPE-Conv saw their BT increase by a mean of 0.24 °C, while those who used PPE-PAPR saw it reduce by a mean of 0.3 °C. 

The differences in temperature registered by the surface camera in the facial area experienced a decrease with both types of PPE (PPE-Conv = 0.19 °C and PPE-PAPR = 0.17 °C). Relevant differences were observed in the differentials of surface temperature at the body level: body temperature increased 0.54 °C in PPE-Conv and decreased 0.33 °C in PPE-PAPR.

**Figure 8:**
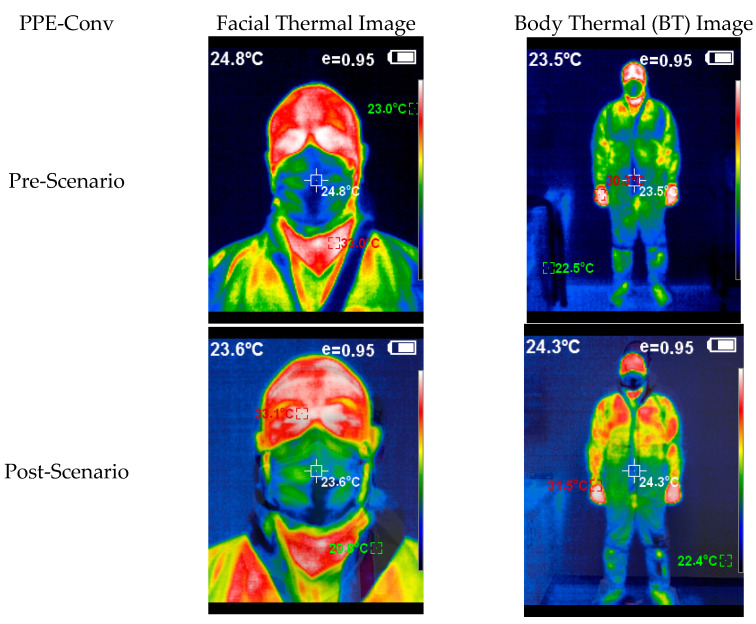
Thermal image subject PPE-Conv pre–post.

**Figure 9:**
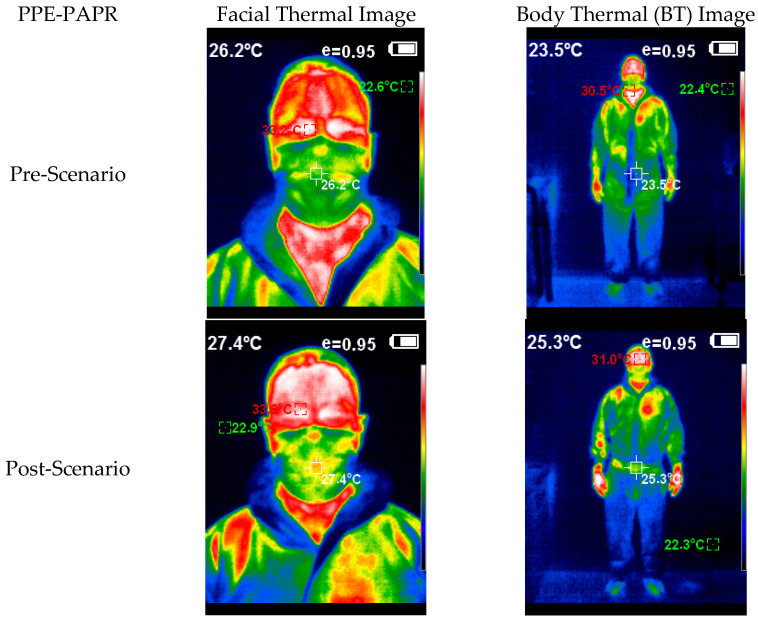
Thermal image subject PPE-PAPR pre–post.

In the images below (Figure 8 and Figure 9), we can see an example of the thermal images taken after the activity of a studied team. Note the thermal image change after the experiment, and how the subject with PPE-Conv has a facial and body thermal image showing areas of greater heat (colored in warm orange-red colors) than the subjects who used PPE-PAPR.

### 3.3. Bivariate Analysis

A bivariate analysis was made between the main variables (BMI, weight loss, percentage weight loss, general heat, facial heat and perspiration) in both samples (PPE-Conv vs. PPE-PAPR).

Given the asymmetric properties of the samples, Spearman’s rank correlation coefficient (*r*_s_) was chosen to determine the relationship between the variables, accepting as relational hypotheses those values with *p*-values of <0.05. The results of the Spearman matrix are shown in Table 8 (PPE-Conv) and Table 9 (PPE-PAPR).

There was no bivariate relationship between the temperature parameters and the rest of the variables related to weight, for either PPE. 

## 4. Discussion

The results showed that the subjects who utilized PPE, whatever the type, suffered a loss of weight (a mean of 200 g in all users). This weight loss was partly measured by the weight gain of the joint PPE used, as can be seen in the relationship between PW loss and the differential of PPE-W. These data were statistically significant according to the paired *t*-test. 

The percentage of body weight lost by the participants was below the limits given for clinical dehydration. These limits are established in differentials between 1% and 3% of body weight [10,13]. This percentage in subjects who used PPE for COVID-19 was similar for both groups (around 0.28–0.29% of initial weight). These figures are far from the ratios given for clinical dehydration. 

With the activity and time constraints of our experiment, the losses experienced by the participants were close to those recorded for elite sports [19] such as badminton [20], water polo [21], and futsal [22,23], with percentages of body mass lost similar to those obtained in our study (between 0.3% and 0.4% of weight of the participating athletes).

If we attend to the sensations reported by the participants relative to sweating, we can see that these changes in the weights are due to the sweating of the participants; this sweat is retained by the garments, so it is not evaporated to the outside. 

Thus, PPE wearers behave in a similar manner to athletes during intense sports practices that usually produce significant changes in body weight, mainly caused by the loss of water in the form of sweat [19].

The sensation of perspiration is a constant finding in the results from measuring the impact of PPE on health workers. Our findings point in the same direction: the use of PPE commonly used in a critical COVID-19 environment implies a high sensation of sweating, which translates into the dampness of the clothes that are worn.

Jacklitsch et al. [24,25] indicated that when the “extent of skin wetted with sweat approaches 20%, the sensation of discomfort begins to be noted. Discomfort is marked and performance decrements can appear with between 20% and 40% wetting of the body surface; performance decrements become increasingly noted as approaches 60%. Sweat begins to be wasted, dripping rather than evaporating at 70%; physiologic strain becomes marked between 60% and 80%. Increases above 80% result in limited tolerance, even for physically fit, heat-acclimatized young workers.” 

Factors such as exercise intensity, body mass, the use of protective uniforms, and hot-humid environments, as well as factors that limit the possibility of replacing fluids (low fluid availability or opportunity for drinking breaks) are related to situations of high water loss (hypohydration) [26].

These situations were identified in our study, especially those that involved the use of uniforms that prevented heat loss, the presence of a hot-humid environment (understanding the interior of the PPE as a “microclimate” with a high temperature and high humidity), and the inability to replace fluids during the caregiving activity. 

In this sense, the use of protective equipment increases the temperature of the body surface, and the sensation of heat and perspiration, as shown by different investigations [27,28,29], conditions the appearance of thermal stress in PPE users. Our research shows that both types of PPE cause an increase in the sensation of heat; however, it was higher in the PPE-Conv users than the PPE-PAPR users. 

Regarding the temperature determinations in the participants, the average of the differential of the temperatures recorded in the three different points (temple, face, and body) was lower in the users of the PAPR equipment than in the others; the highest differential temperature was registered in the users of conventional equipment. This could justify the greater comfort indicated by the users of PPE-PAPR over PAPR-Conv, and could indicate the positive effect of the ventilation systems and the air flow of this equipment.

The body surface temperature and the temple temperature increased in users of PPE-Conv, but did not imply a risk for their health. This situation can be explained in part by the adequate homoeothermic reaction, with the production of sweat for an adequate loss of surface heat and increased sensations of facial and body heat.

If these compensatory mechanisms are not present and adequate sweating does not occur, the subject could incur a failure in homoeothermic ability and an increase in core temperature, with a consequent risk to health.

Maintaining a flow of air, either inside (PPE-PAPR-in) the PPE or on its surface (PPE-PAPR-out), contributes to reducing the internal heat footprint, which in turn improves perspiration and the sensation experienced by those who use it. The use of this type of PPE could avoid the thermal discomfort effect and the negative consequence of thermal stress [30,31,32,33,34].

Regarding the length of time performing an activity, it was much longer in a critical environment as compared to longer-lasting sports activities (240 vs. 90–120 min), as evidenced in previous studies [8] and in the data collected in the present study. Another differential factor was the intensity of the activity, where the sporting activity logically implies physical efforts that are more intensive than the activities commonly performed in clinical settings, except for situations such as caring for amyotrophic lateral syndrome (ALS) patients.

The combination of heat/humidity situations, along with physical effort, prolonged time, and high rates of sweating, could condition the appearance of negative effects such as cognitive impairment, impaired decision making, mental fatigue, decreased ability to concentrate, and impaired physical ability. The repercussions are greater as the loss of hydration increases [19,35,36]. The loss of body water with sweating during prolonged heat exposure and/or exercise leads to dehydration if fluid replacement is insufficient to match the rates of fluid loss. 

This evidence indicates that these negative manifestations of hypohydration–dehydration are not homogeneous, and therefore, each person has a different amount of resilience, “compensating” for these negative manifestations by increasing the “effort” made to successfully complete the tasks assigned to them.

The sensation of thirst is useful for determining the need for fluid intake during daily life, but it is relatively insensitive for acute monitoring of the state of thirst and hydration during exercise [37]. For this reason, it is not a good indicator of the state of hypohydration of an individual [38]. 

All of users of PPE for COVID-19 had an increased thirst differential, regardless of the PPE used and the degree of fluid loss observed, in agreement with previous studies [39,40].

When the effects of dehydration–hypohydration are considered, different studies have indicated the pattern of replacement of lost fluid. The evidence in this field recommends hydration strategies maintained over time. Some authors [36,37] have indicated short periods of time (15–20 min) between fluid intake events and the intake of liquids at will to replace lost liquids after exercises, even if one is not thirsty. The intake of large amounts of fluids prior to a physical activity event with possible sweating can induce situations of hypervolemic hyponatremia, as seen in endurance athletes who have a large intake of fluids before an endurance exercise event, so this practice is discouraged [38]. Hydration guidelines are recommended with moderate volumes at particular fractions of time.

However, the amount of volume to be ingested as a guideline for hydration during and after intense exercise is related to the amount of volume that is lost during activity; and since we do not know the total volume lost in a four-hour activity as it would be in a real clinical environment, we cannot indicate an exact amount of fluid to replace or a hydration pattern to follow. We simply advise drinking water before feeling thirsty.

Despite the presence of the same physical repercussions associated with the loss of water and the increase in the sensation of thirst, the degree of comfort of the PPE associated with the thermal sensation experienced both at the general and facial levels, as well as the sensation of perspiration, was better with the PAPR equipment than with the conventional PPE, especially when these were used inside the suit, confirming the results from previous studies [39,40]. This type of use adds a ventilation effect for the torso, which, although unable to mitigate sweating, contributes to a better feeling of general comfort. This also explains why the PPE removed from these individuals accumulated less weight from the sweat retained in the garments: the ventilation generated contributed to evaporation of part of the sweat accumulated in the garment, thereby improving the feeling of wellbeing. Nevertheless, the use of PAPR represents an added complication regarding mobility and capacity to communicate and requires training for its safe handling by the worker [41]. 

International and occupational organizations, (Public Heath England; The National Institute for Occupational Safety and Health/NIOSH-USA) recognize the danger posed to the health of workers using PPE in relation to dehydration and heat stress, issuing general and specific recommendations in relation to this field. Ti et al. [42] recommended the routine use of PAPR during the induction and reversal of anesthesia for all personnel within 2 m of the patient, at all times during airway instrumentation, and for the transport of critically ill patients.

Respiratory protection for healthcare workers should not wait for definitive scientific evidence in circumstances of emerging lethal diseases, but should focus on optimal prevention tailored to the situation.

### Limitations

The research findings cannot be generalized because of the small sample size. The character of the pilot study and the low number of participants are limitations to consider, limiting the internal and external validity of our data, but they serve as the basis for further studies. 

Another factor to consider is the absence of a control group in the study and expected duration of the simulated situations, since these are far from the times given in real environments. This factor limits the results obtained and, thus, limits the validity of the conclusions. 

The observed weight losses were after a greater than 45 min exposure time to the activity; activity times in real environments range to over four hours, so determining the risk of dehydration is a hypothesis that requires measurements in real environments, after the removal of PPE by healthcare workers.

This study piloted the methodology to carry out these studies in real environments.

## 5. Conclusions

The use of PPE in the management of critically ill COVID-19 patients generated weight loss related to excessive sweating. The weight loss shown in this pilot test was far from the clinical limits of dehydration.

The use of ventilated PPE, such as PAPR, reduced the body temperature and heat sensation experienced by its users, at the same time improving the comfort of those wearing it.

The simulated environment is a suitable place to develop the piloting of applicable research methodologies in future studies in a real environment.

## Figures and Tables

**Figure 2:**
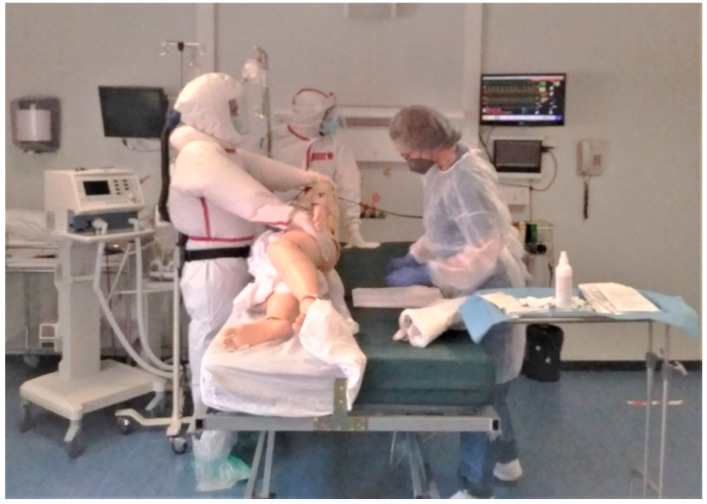
Subject during a simulated scenario. RN and HCA during basic care.

**Figure 3:**
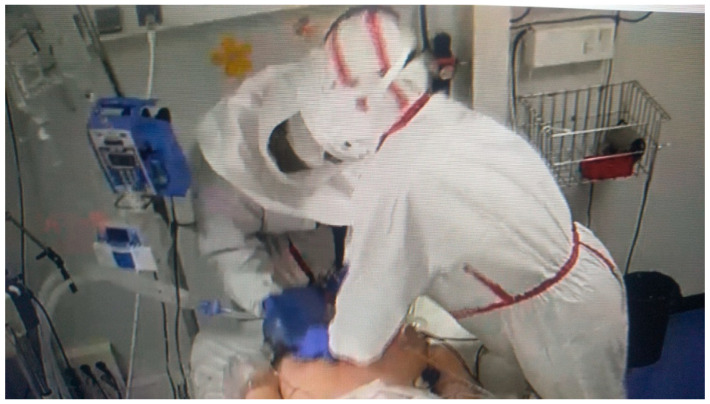
RN during a simulated scenario. Advanced life support activity.

**Table 1:** Description of the activity planed in the simulated scenario.

Basic Care (Mild Physical Activity) (Figure 2)	Advanced Life Support Care (High Physical Activity) (Figure 3)
Hygiene of the patient; oral hygiene-orotracheal tube, according to the Spanish Society of Intensive and Critical Medicine and Coronary Units (SEMICYUC) Pneumonia Zero protocol; cure and care of venous (peripheral and central) and arterial catheters, according to Bacteremia Zero protocols; change of fluid therapy systems; venous and arterial blood sample extraction; extraction and culture of bronchial secretions; performance of routine electrocardiogram; and adjustment of infusions and change of pump systems.	Advanced life support, according to the European Resuscitation Council (ERC-2020) regulations, on resuscitation in COVID-19 patients: defibrillation, external cardiac massage, and administration of drugs.

**Table 2:** Description of the PPE used in the experiment.

	PPE-Conventional	PPE-PAPR
Body Protection (Joint Equipment)	Disposable soft surgical medical uniforms. Medline P35PBL—35 g/m^2^ CoverStar Plus. Disposable full-body suit, made of 100% polypropylene material, with sealed seams and an elasticated hood, cuffs, and ankles, with certified protection against particles, liquid splashes, and low-pressure aerosols (cat III type 4/5/6). CAT III Type 4B/5B/6B. 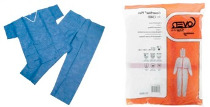	Disposable soft surgical medical uniforms. Medline P35PBL—35 g/m^2^ CoverStar Plus. Disposable full-body suit, made of 100% polypropylene material, with sealed seams and an elasticated hood, cuffs, and ankles, with certified protection against particles, liquid splashes, and low-pressure aerosols (cat III type 4/5/6). CAT III Type 4B/5B/6B 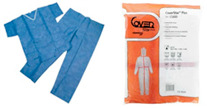
Respiratory Protection	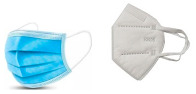 Respiratory protection mask FFP2-KN95 without an exhalation valve, and a surgical mask over it.	Versaflow 3M equipment, composed of: -3M™ S-655 hood with head suspension and coverage for head, face, neck, and shoulders; -3M TR-302E ventilator, Versaflow TR-300 series, with nozzle and filter 3M™ Versaflow™ TR-3712E certified FFP3 protection. With a combined weight of 1.8 kg. 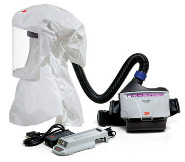
Facial Protection	Face shield (full-face protection screen with a foam band on the forehead to absorb sweat and prevent chafing and to provide enough space for optical or safety glasses) and safety glasses (wide pressure tape, with indirect ventilation that improves air circulation and reduces fogging in hot/humid conditions). 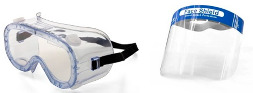	
Hand Protection	Double gloves: sterile gloves over nitrile gloves.	Double gloves: sterile gloves over nitrile gloves.
Example Subjects: Joint Protective Equipment	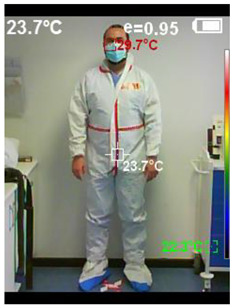	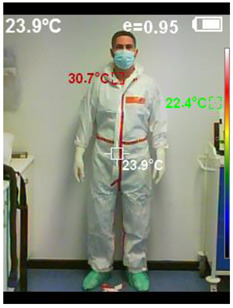

**Table 3:** Experimental protocol.

RECEPTION (15 min)	ACCLIMATION (10–15 min)			SIMULATED SCENARIO (45–60 min)	CLOSURE-RE-HYDRATION (15–20 min)
Reception of the Participants	Situation Briefing	PPE Fitting	Initial Checks	Simulated Scene	Sim Room Exit
Exchange of impressions. Exhibition of work plan. Change of clothes. Putting on paper medical uniforms. Initial weighing. Determination of thirst	Explanation of the environment. Show arrangement and location of resources.	Conventional PPE vs. EPP PAPR	Determination of the physical conditions of the room (Tª, humidity, and noise).	Basic care (30 min minimum). Complete patient hygiene; change sheets; mouth hygiene and TOT; care of catheters and wounds; administration of medication; calculation of water balance; conducting additional tests.	Removal of used PPE and soft surgical medical uniforms. Putting on new dry soft surgical medical uniforms. Final weighing. Wet PPE and soft surgical medical uniform weighing. Determination of thirst. Register self-administered scales. Rehydration and exchange of impressions.
				SVA care (15 min max.). Realization of MCE; defibrillation and administration of medication ordered via Tlf.	

**Table 4:** Sociodemographic characteristics.

	*n*	%	Mean ± SD	Min.–Max.
Age			35 ± 5.86	(44–24)
Gender				
Male	4	25.0		
Female	12	75.0		
BMI			23.7 ± 3.10	(20.6–32.7)
Male BMI			27.67 ± 3.11	32.7–25.6
Female BMI			22.37 ± 1.72	25.6–20.6
Experience				
<6 months	6	37.5		
6–12 months	5	12.5		
1–2 years	1	6.3		
>2 years	7	43.8		
Type PPE used in a real setting				
PPE-Conv	16	100		
PPE-PAPR	4	25		
Max. PPE time in a real setting			3.75 ± 0.86	(2–5)

**Table 5:** Weight and comfort parameters.

	Descriptive Statistics		Normality Measures		
	Mean ± SD	Min.–Max.	Skewness (Error Tip)	Kurtosis (Error Tip)	Shapiro–Wilk (Sig.) > 0.05
BMI (kg/m^2^)					
PPE-Conv (*n* = 16)	23.7 ± 3.02	20.6–32.7	1.72 (0.564)	3.826 (1.091)	0.834 (0.008)
PPE-PAPR (*n* = 16)	23.7 ± 3.01	20.6–32.8	1.79 (0.564)	4.02 (1.091)	0.824 (0.006)
Differential Personal Weight (PW) Before/After (kg)					
PPE-Conv (*n* = 16)	0.200 ± 0.13	0.1–0.4	0.90 (0.564)	0.96 (1.091)	0.902 (0.086)
PPE-PAPR (*n* = 16)	0.206 ± 0.11	0.1–0.4	0.83 (0.564)	−0.54 (1.091)	0.800 (0.003)
% Lost Weight					
PPE-Conv (*n* = 16)	0.28 ± 0.13	0–0.46	−0.55 (0.564)	−0.74 (1.091)	0.925 (0.205)
PPE-PAPR (*n* = 16)	0.29 ± 0.12	0.15–0.53	0.56 (0.564)	(−0.75) (1.091)	0.896 (0.068)
Differential BMI Before/After (kg/m^2^)					
PPE-Conv (*n* = 16)	–0.068 ± 0.04	–0.13 to 0	−0.102 (0.564)	0.336 (1.091)	0.956 (0.585)
PPE-PAPR (*n* = 16)	–0.07 ± 0.03	–0.13 to –0.03	−0.613 (0.564)	−0.857 (1.091)	0.885 (0.046)
Differential Weight PPE (PPE-W) Before/After (g)					
PPE-Conv (*n* = 16)	75.75 ± 57.62	0–186	0.285 (0.564)	−0.22 (1.091)	0.914 (0.134)
PPE-PAPR (*n* = 16)	65.06 ± 45.96	8–165	0.99 (0.564)	0.56 (1.091)	0.896 (0.07)
PAPR-out (*n* = 8)	83.13 ± 33.4	52–157			
PAPR-in (*n* = 8)	47.0 ± 51.64	8–165			
Differential Thirst Before/After (0 = Min./5 = Max.)					
PPE-Conv (*n* = 16)	2.56 ±1.09	1–5	0.692 (0.564)	0.23 (1.091)	
PPE-PAPR (*n* = 16)	2.44 ± 0.73	1–4	0.25 (0.564)	0.25 (1.091)	
General Heat Sensation (0 = Worst/5 = Best)					
PPE-Conv (*n* = 16)	1.44 ± 1.75	0–5	1.27 (0.564)	0.45 (1.091)	
PPE-PAPR (*n* = 16)	2.94 ± 1.61	0–5	−1.09 (0.564)	0.05 (1.091)	
Facial Heat Sensation (0 = Worst/5 = Best)					
PPE-Conv (*n* = 16)	1.01 ± 1.932	0–5	0.95 (0.564)	−0.714 (1.091)	
PPE-PAPR (*n* = 16)	3.19 ± 1.60	0–5	−1.02 (0.564)	0.27 (1.091)	
Sensation of Perspiration (0 = Min./5 = Max.)					
PPE-Conv (*n* = 16)	4.56 ± 1.26	0–5	−3.56 (0.564)	13.27 (1.091)	
PPE-PAPR (*n* = 16)	3.44 ± 1.21	1–5	−0.48 (0.564)	(−0.60 (1.091)	

**Table 6:** Paired *t*-test for personal weight and joint PPE.

			Confidence Interval for the Difference		
Paired *t*-Test PW	Mean ± SD	Standard Error of the Mean	Lower	Higher	Sig. (Bilateral)
PW after–PW before PPE-Conv (*n* = 16)	0.2000 ± 0.126	0.03162	0.13260	0.26740	0.000
PW after–PW before PPE-PAPR (*n* = 16)	0.20625 ± 0.112	0.02809	0.14638	0. 26612	0.000
Paired *t*-Test JPEW					
JPEW after–JPEW before PPE-Conv (*n* = 16)	−75.75 ± 57.626	14.41	−106.46	−45.04	0.000
JPEW after–JPEW before PPE-PAPR (*n* = 16)	−65.063 ± 45.96	11.49	−89.55	−40.57	0.000

**Table 7:** Temperture parameters.

	Descriptive Statistics		Normality Measures		
	Mean ± SD	Min.–Max.	Skewness (Error Tip)	Kurtosis (Error Tip)	Shapiro–Wilk (Sig.) > 0.05
Temple Temperature Before/After (°C)					
PPE-Conv (*n* = 16)	36.51 ± 0.21	35.8–36.8			
PPE-PAPR (*n* = 16)	36.4 ± 0.26	35.6–36.4			
Differential Temple Temperature Before/After (°C)					
PPE-Conv (*n* = 16)	0.237 ± 0.24	−0.3 to 0.6	−0.606 (0.564)	−0.314 (1.091)	0.946 (0.422)
PPE-PAPR (*n* = 16)	−0.29 ± 0.18	−0.6 to 0.1	−0.453 (0.564)	−0.883 (1.091)	0.911 (0.12)
Facial Temperature Before/After (°C)					
PPE-Conv (*n* = 16)	33.85 ± 0.77	32.0–35.0			
PPE-PAPR (*n* = 16)	33.80 ± 0.95	32.3–35.6			
Differential Facial Temperature Before/After (°C)					
PPE-Conv (*n* = 16)	−0.13 ± 0.71	−1.5 to 1.2	−0.057 (0.564)	−0.570 (1.091)	0.971 (0.847)
PPE-PAPR (*n* = 16)	−0.17 ± 0.75	−2 to 0.9	−0.883 (0.564)	1.221 (1.091)	0.941 (0.360)
Body Temperature Before/After (°C)					
PPE-Conv (*n* = 16)	32.38 ± 1.33	30.5–34.5			
PPE-PAPR (*n* = 16)	31.04 ± 1.27	28.9–34.0			
Differential Body Temperature Before/After (°C)					
PPE-Conv (*n* = 16)	0.54 ± 0.74	−1.1 to 1.7	−0.448 (0.564)	0.201 (1.091)	0.974 (0.898)
PPE-PAPR (*n* = 16)	−0.33 ± 1.3	−2.9 to 1.8	−0.637 (0.564)	0.67 (1.091)	0.922 (0.184)

**Table 8:** Rho Spearman. PPE-CONV.

(Sig. < 0.05)	DIF. WEIGH	% LOSS	GEN. HEAT	FAC. HEAT	SENS. PERSP	DIF. THIRST	DIF.–WEIGH PPE
BMI	0.33 (0.21)	0.07 (0.79)	−0.13 (0.63)	−0.13 (0.63)	−0.37 (0.159)	0.08 (0.97)	0.544 (0.029)
DIF. WEIGH		0.949 (0.000)	−0.172 (0.52)	0.012 (0.97)	0.078 (0.078)	0.486 (0.056)	0.510 (0.044)
% LOSS			−0.062 (0.82)	0.065 (0.81)	0.270 (0.31)	0.464 (0.07)	0.460 (0.073)
GEN. HEAT				0.563 (0.023)	0.051 (0.852)	−0.24 (0.37)	−0.377 (0.150)
FAC. HEAT					0.028 (0.919)	−0.11 (0.68)	−0.097 (0.72)
SENS. PERSP						−0.263 (0.325)	0.121 (0.656)
DIF. THIRST							0.250 (0.35)

**Table 9:** Rho Spearman. PPE-PAPR.

(Sig. < 0.05)	DIF. WEIGH	% LOSS	GEN. HEAT	FAC. HEAT	SENS. PERSP	DIF. THIRST	DIF.–WEIGH PPE
BMI	0.39 (0.126)	0.25 (0.93)	−0.351 (0.18)	−0.259 (0.33)	0.45 (0.08)	−0.15 (0.58)	0.29 (0.28)
DIF. WEIGH		0.912 (0.000)	−0.139 (0.608)	−0.268 (0.316)	−0.122 (0.653)	0.377 (0.150)	0.245 (0.361)
% LOSS			−0.044 (0.87)	−0.255 (0.340)	0.098 (0.718)	0.467 (0.068)	0.205 (0.446)
GEN. HEAT				0.411 (0.11)	0.08 (0.77)	−0.167 (0.537)	−0.267 (317)
FAC. HEAT					−0.38 (0.146)	−0.571 (0.021)	−0.473 (0.063)
SENS. PERSP						0.357 (0.174)	0.163 (0.546)
DIF. THIRST							0.254 (0.342)

## Data Availability

The data are available upon email request to the corresponding authors.

## References

[1] Arabi Y.M., Azoulay E., Al-Dorzi H.M., Phua J., Salluh J., Binnie A., Hodgson C., Angus D.C., Cecconi M., Du B. (2021). How the COVID-19 pandemic will change the future of critical care. Intensive Care Med..

[2] El-Boghdadly K., Wong D.J.N., Owen R., Neuman M.D., Pocock S., Carlisle J.B., Johnstone C., Andruszkiewicz P., Baker P.A., Biccard B.M. (2020). Risks to healthcare workers following tracheal intubation of patients with COVID-19: A prospective international multicentre cohort study. Anaesthesia.

[3] Cook T.M., El-Boghdadly K., McGuire B., McNarry A.F., Patel A., Higgs A. (2020). Consensus guidelines for managing the airway in patients with COVID-19. Anaesthesia.

[4] Chu D.K., Akl E.A., Duda S., Solo K., Yaacoub S., Schünemann H.J., Chu D.K., Akl E.A., El-harakeh A., Bognanni A. (2020). Physical distancing, face masks, and eye protection to prevent person-to-person transmission of SARS-CoV-2 and COVID-19: A systematic review and meta-analysis. Lancet.

[5] Guidance for Wearing and Removing Personal Protective Equipment in Healthcare Settings for the Care of Patients with Suspected or Confirmed COVID-19. https://www.ecdc.europa.eu/en/publications-data/guidance-wearing-and-removing-personal-protective-equipment-healthcare-settings.

[6] Atay S., Cura Ş.Ü. (2020). Problems Encountered by Nurses Due to the Use of Personal Protective Equipment During the Coronavirus Pandemic: Results of a Survey. Wound Manag. Prev..

[7] Ong J.J., Bharatendu C., Goh Y., Tang J.Z., Sooi K.W., Tan Y.L., Tan B.Y., Teoh H.L., Ong S.T., Allen D.M. (2020). Headaches Associated With Personal Protective Equipment—A Cross-Sectional Study Among Frontline Healthcare Workers During COVID-19. Headache J. Head Face Pain.

[8] Dhandapani M., Jose S., Cyriac M.C. (2021). Health Problems and Skin Damages Caused by Personal Protective Equipment: Experience of Frontline Nurses Caring for Critical COVID-19 Patients in Intensive Care Units. Indian J. Crit. Care Med..

[9] Jiang Q., Liu Y., Wei W., Zhu D., Chen A., Liu H., Wang J., Jiang Z., Han Q., Bai Y. (2020). The prevalence, characteristics, and related factors of pressure injury in medical staff wearing personal protective equipment against COVID-19 in China: A multicentre cross-sectional survey. Int. Wound J..

[10] Cheuvront S.N., Kenefick R.W. (2017). CORP: Improving the status quo for measuring whole body sweat losses. J. Appl. Physiol..

[11] Fernández-Méndez M., Otero-Agra M., Fernández-Méndez F., Martínez-Isasi S., Santos-Folgar M., Barcala-Furelos R., Rodríguez-Núñez A. (2021). Analysis of Physiological Response during Cardiopulmonary Resuscitation with Personal Protective Equipment: A Randomized Crossover Study. Int. J. Environ. Res. Public Health.

[12] Rauch S., van Veelen M.J., Oberhammer R., Cappello T.D., Roveri G., Gruber E., Strapazzon G. (2021). Effect of Wearing Personal Protective Equipment (PPE) on CPR Quality in Times of the COVID-19 Pandemic—A Simulation, Randomised Crossover Trial. J. Clin. Med..

[13] Harvey G., Meir R., Brooks L., Holloway K. (2008). The use of body mass changes as a practical measure of dehydration in team sports. J. Sci. Med. Sport.

[14] MacLeod H., Cooper S., Bandelow S., Malcolm R., Sunderland C. (2018). Effects of heat stress and dehydration on cognitive function in elite female field hockey players. BMC Sports Sci. Med. Rehabil..

[15] Zhang N., Du S.M., Zhang J.F., Ma G.S. (2019). Effects of Dehydration and Rehydration on Cognitive Performance and Mood among Male College Students in Cangzhou, China: A Self-Controlled Trial. Int. J. Environ. Res. Public Health.

[16] Roussin C.J., Weinstock P. (2017). SimZones: An Organizational Innovation for Simulation Programs and Centers. Acad. Med..

[17] Schumacher J., Arlidge J., Garnham F., Ahmad I. (2017). A randomised crossover simulation study comparing the impact of chemical, biological, radiological or nuclear substance personal protection equipment on the performance of advanced life support interventions. Anaesthesia.

[18] Schumacher J., Arlidge J., Dudley D., Sicinski M., Ahmad I. (2020). The impact of respiratory protective equipment on difficult airway management: A randomised, crossover, simulation study. Anaesthesia.

[19] Nuccio R.P., Barnes K.A., Carter J.M., Baker L.B. (2017). Fluid Balance in Team Sport Athletes and the Effect of Hypohydration on Cognitive, Technical, and Physical Performance. Sports Med..

[20] Abián-Vicén J., Coso J.D., González-Millán C., Salinero J.J., Abián P. (2012). Analysis of Dehydration and Strength in Elite Badminton Players. PLoS ONE.

[21] Cox G.R., Broad E.M., Riley M.D., Burke L.M. (2002). Body mass changes and voluntary fluid intakes of elite level water polo players and swimmers. J. Sci. Med. Sport.

[22] Perrone C., Sehl P., Martins J.B., Meyer F. (2011). Hydration Status and Sweating Responses of Boys Playing Soccer and Futsal. Med. Sport..

[23] Fortes L.S., Nascimento-Júnior J.R., Mortatti A.L., Lima-Júnior D.R., Ferreira M.E. (2018). Effect of Dehydration on Passing Decision Making in Soccer Athletes. Res. Q. Exerc. Sport.

[24] Jacklitsch B.L., Williams W.J., Musolin K., Coca A., Kim J.H., Turner N. (2016). Criteria for a Recommended Standard: Occupational Exposure to Heat and Hot Environments.

[25] Jacklitsch B.L., Williams W.J., Musolin K., Coca A., Kim J.H., Turner N. (2016). Occupational Exposure to Heat and Hot Environments. Revised Criteria 2016.

[26] Lee J., Venugopal V., Latha P.K., Alhadad S.B., Leow C.H.W., Goh N.Y.D., Tan E., Kjellstrom T., Morabito M., Lee J.K.W. (2020). Heat Stress and Thermal Perception amongst Healthcare Workers during the COVID-19 Pandemic in India and Singapore. Int. J. Environ. Res. Public Health.

[27] Davey S.L., Lee B.J., Robbins T., Randeva H., Thake C.D. (2021). Heat stress and PPE during COVID-19: Impact on healthcare workers’ performance, safety and well-being in NHS settings. J. Hosp. Infect..

[28] Messeri A., Bonafede M., Pietrafesa E., Pinto I., de’Donato F., Crisci A., Lee J.K.W., Marinaccio A., Levi M., Morabito M. (2021). A Web Survey to Evaluate the Thermal Stress Associated with Personal Protective Equipment among Healthcare Workers during the COVID-19 Pandemic in Italy. Int. J. Environ. Res. Public Health.

[29] López-Sánchez J.I., Hancock P.A. (2018). Thermal effects on cognition: A new quantitative synthesis. Int. J. Hyperth..

[30] Luze H., Nischwitz S.P., Kotzbeck P., Fink J., Holzer J.C.J., Popp D., Kamolz L.-P. (2021). Personal protective equipment in the COVID-19 pandemic and the use of cooling-wear as alleviator of thermal stress. Wien. Klin. Wochenschr..

[31] Kenny G.P., Flouris A.D., Wang F., Gao C. (2014). The human thermoregulatory system and its response to thermal stress. Protective Clothing.

[32] Flouris A.D., Dinas P.C., Ioannou L.G., Nybo L., Havenith G., Kenny G.P., Kjellstrom T. (2018). Workers’ health and productivity under occupational heat strain: A systematic review and meta-analysis. Lancet Planet. Health.

[33] Havenith G., den Hartog E., Martini S. (2011). Heat stress in chemical protective clothing: Porosity and vapour resistance. Ergonomics.

[34] Adams W.M., Vandermark L.W., Belval L.N., Casa D.J. (2019). The Utility of Thirst as a Measure of Hydration Status Following Exercise-Induced Dehydration. Nutrients.

[35] Carroll H.A., Chen Y.-C., Templeman I., James L.J., Betts J.A., Trim W.V. (2020). The effect of hydration status on plasma FGF21 concentrations in humans: A subanalysis of a randomised crossover trial. PLoS ONE.

[36] Kenefick R.W. (2019). Author’s Reply to Goulet: Comment on: “Drinking Strategies: Planned Drinking Versus Drinking to Thirst”. Sports Med..

[37] Gantois P., Ferreira M.E.C., Lima-Junior D.D., Nakamura F.Y., Batista G.R., Fonseca F.S., Fortes L.D.S. (2020). Effects of mental fatigue on passing decision-making performance in professional soccer athletes. Eur. J. Sport Sci..

[38] Hew-Butler T., Ayus J.C., Kipps C., Maughan R.J., Mettler S., Meeuwisse W.H., Page A.J., Reid S.A., Rehrer N.J., Roberts W.O. (2008). Statement of the Second International Exercise-Associated Hyponatremia Consensus Development Conference, New Zealand, 2007. Clin. J. Sport Med..

[39] Licina A., Silvers A., Stuart R.L. (2020). Use of powered air-purifying respirator (PAPR) by healthcare workers for preventing highly infectious viral diseases—a systematic review of evidence. Syst. Rev..

[40] Licina A., Silvers A. (2021). Use of powered air-purifying respirator(PAPR) as part of protective equipment against SARS-CoV-2-a narrative review and critical appraisal of evidence. Am. J. Infect. Control.

[41] Verbeek J.H., Rajamaki B., Ijaz S., Tikka C., Ruotsalainen J.H., Edmond M.B., Sauni R., Balci F.S.K. (2020). Personal protective equipment for preventing highly infectious diseases due to exposure to contaminated body fluids in healthcare staff. Cochrane Database Syst. Rev..

[42] Ti L.K., Ang L.S., Foong T.W., Ng B.S.W. (2020). What we do when a COVID-19 patient needs an operation: Operating room preparation and guidance. Can. J. Anesth. Can. Anesth..

